# Strategies of Endoscopic Management of Upper Tract Urothelial Carcinoma among Endourologists: A Global Survey

**DOI:** 10.3390/jpm13040591

**Published:** 2023-03-28

**Authors:** Asaf Shvero, Orel Carmona, Dorit E. Zilberman, Zohar A. Dotan, Miki Haifler, Nir Kleinmann

**Affiliations:** 1 The Department of Urology, Sheba Medical Center, Ramat Gan 5262000, Israel; orel.carmona@sheba.health.gov.il (O.C.); dorit.zilberman@sheba.health.gov.il (D.E.Z.); zohar.dotan@sheba.health.gov.il (Z.A.D.); michael.haifler@sheba.health.gov.il (M.H.); nir.kleinmann@sheba.health.gov.il (N.K.); 2 The Sackler School of Medicine, Tel Aviv University, Tel Aviv 6997801, Israel

**Keywords:** upper tract urothelial carcinoma, ureteroscopy, nephron-sparing surgery, laser surgery

## Abstract

Up-to-date guidelines on the management of upper tract urothelial carcinoma (UTUC) are continuously published. We aim to assess the variability of diagnosis and treatment strategies in the endoscopic management of UTUC and adherence to European Association of Urology and National Comprehensive Cancer Network guidelines. A 15-question survey was designed to query practitioners on approaches to clinical practice and knowledge about endoscopic treatment indications and techniques. It was emailed to all members of the Endourologic Society through the society’s office, and to all Israeli non-member endourologists. Eighty-eight urologists participated in the survey. Adherence to guidelines on indications for endoscopic management was only 51%. Most of the survey respondents (87.5%) use holmium laser for tumor ablation, and ~50% use forceps for biopsy while the other half use baskets. Only 50% stated that they would use Jelmyto^®^ for specific indications. Most (80%) indicated that they repeat the ureteroscopy 3 months after the first one, and 52.3% continue with follow-up ureteroscopy every 3 months during the first year after diagnosis. There is vast variability among endourologists in the technical aspects of UTUC, the indications for endoscopic management, and adherence to the available guidelines for managing UTUC.

## 1. Introduction

The gold standard therapy for UTUC is nephroureterectomy with bladder cuff excision [1], but a solitary kidney status may induce renal insufficiency and lead to higher rates of dialysis, cardiovascular morbidity, and overall mortality [2,3,4,5]. This might rule out nephroureterectomy as the treatment of choice for patients with a solitary kidney, bilateral tumors, chronic renal failure, and those with a high surgical risk. In addition, radical nephroureterectomy has been associated with a significant complications rate of almost 10% major complications [6,7,8]. These considerations led to the development of kidney-sparing therapies, such as segmental ureterectomy and endoscopic treatments. Endoscopic treatments for UTUC have been made possible with advances in endoscopic optical and deflection technologies and have gained popularity worldwide [9,10]. The National Comprehensive Cancer Network (NCCN) published recommendations on the management of UTUC according to which non-metastatic, noninvasive, and low-grade relatively small tumors are favorable for endoscopic treatment [11]. The European Association of Urology (EAU) developed a risk-adapted protocol for kidney-sparing management, classifying patients with low-grade and low-volume tumors as having low-risk UTUC, making them suitable for endoscopic management [10,12]. However, the NCCN and EAU definitions of low-risk UTUC have been challenged over the past few years. For example, while the EAU guidelines used to refer to tumors larger than 1 cm as “high-risk UTUC”, recent publications have shown feasibility and good oncological outcomes in the treatment of even larger tumors [13,14,15,16,17]. In addition, the advances in laser technology [18,19,20], as well as the refinement of biopsy methods [21,22], have widened the armamentarium of the endourologist in accurately diagnosing and treating UTUC. In addition, a topical mitomycin-containing hydrogel Jelmyto^®^ was proven to be an effective ablative agent for low-grade UTUC [23]. Lastly, cost-analysis studies have shown that from a cost perspective, renal sparing management is effective in reducing health care expenses [24]. We hypothesized that this wealth of choices may also lead to variability in diagnosis and treatment strategies between different surgical centers and among endourologic surgeons. Our goal was to test this hypothesis by examining the variety of approaches to endoscopic treatments of UTUC provided by endourologists from around the world.

## 2. Materials and Methods

The urological literature on the treatment of UTUC was reviewed and data on the endoscopic treatment of UTUC were extracted. A 15-question survey was then designed using a “Google form” application to retrieve approaches to clinical practice and knowledge about controversies regarding endoscopic treatment indications and techniques (Appendix A). The questionnaire was entitled “Upper Urinary Tract Urothelial Carcinoma Management”, and it consisted of multiple choice answers ranging between 2 to 7 yes/no answers. Some questions asked for only one answer out of the several possibilities, while others made it possible to mark several answers. The questions covered the background and professional experience of the respondents, indications for UTUC endoscopic treatment, biopsy and ablation techniques, the use of Jelmyto^®^ topical agent, and follow-up regimens. The questions were tabulated to determine professional background and practice patterns of UTUC endoscopic treatments among endourologists worldwide. The questionnaire contained no means of identification.

The survey was distributed with the assistance of the Endourological Society Office via e-mail to all the Endourological Society members in addition to all fellowship-trained Israeli endourologists who were not members of the Endourological Society. The results were presented as frequency and proportions of the response option to each question. We then looked for an association between the training and experience of the surgeon to the different treatment strategies using logistic regression analysis (binary and nominal according to the dependent variable). For multiple-choice questions, several categories were segmented or united clarification: “Indications for UTUC endoscopic treatment” was broken into “Adherence to grade recommendations”, “Adherence to focality recommendations”, and “Adherence to EAU guidelines”; “When will you use JELLMYTO adjuvant treatment” was narrowed to two option: Use of JELLMYTO and disuse of JELLMYTO. 

## 3. Results

Eighty-eight urologic surgeons participated in the survey. The survey was sent out to an estimated 1300 endourologic society members and Israeli endourologists, yielding a response rate of about 7%. The queries and responses are given in Table 1. 

**Figure 1:**
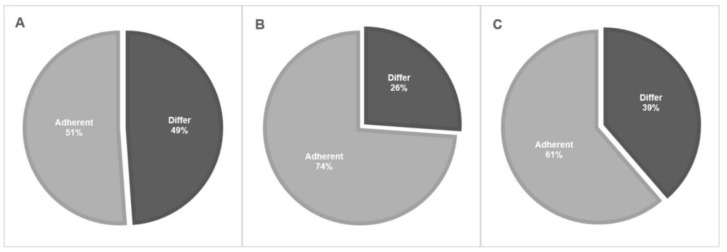
Adherence to EAU and NCCN guidelines on the indications for endoscopic treatment. (**A**)—Total adherence rates. (**B**)—Adherence to tumor grade recommendations. (**C**)—Adherence to focality recommendations.

Most of the respondents (80.7%) were fellowship trained, most of them were endourologists (69%) and most of them (63.5%) had over 10 years of clinical experience since fellowship graduation. Most of the respondents marked low-grade and solitary lesions as indications for endoscopic treatment (92% and 86%, respectively). At the same time, approximately 20% of respondents marked high-grade cytology or pathology as also being suitable for endoscopic treatment. The extent of total adherence to NCCN and EAU guidelines on the indication for endoscopic management is displayed in Figure 1. In total, the adherence rate to indications for endoscopic treatment guidelines was only 51.1%. The most popular energy generator used by 87.5% of our respondents was the Holmium: Yttrium Aluminum Garnet (Ho: YAG) laser. Approximately 50% use forceps for biopsy while the other half use baskets. Only 50% stated that they would use Jelmyto^®^ for specific indications, such as frequent recurrences and a multifocal tumor. The percutaneous approach was very rarely used, with 94.3% of the respondents choosing it for only 0–10% of cases. Only 39.8% of respondents marked tumor-grade progression and 38.6% of them marked high-volume or frequent recurrences as an indication of referral to RNU.

When asked about the endoscopic follow-up protocol for patients with UTUC, 80% indicated they would repeat the ureteroscopy 3 months after the first one, and 52.3% would continue with follow-up ureteroscopy every 3 months during the first year since diagnosis. The cross-sectional imaging follow-up protocol was highly variable, with 41% performing an imaging test every 6 months. All of the survey respondents perform at some point a voiding cytology test, and 51.2% perform a cytology test every 3 months for the first year since diagnosis and then every 6 months.

The association between surgeon training/experience and management patterns of UTUC is given in Table 2. Energy generator use was omitted from the analysis due to an abundance of answers, with many of them being free-text. Table 3 and Table 4 detail the significant associations found.

## 4. Discussion

Our study was the first to assess the variability in the endoscopic management strategies for UTUC between medical centers and surgeons worldwide. Although the NCCN and EAU publish guidelines specific to this pathology, our results revealed that many urologists follow different forms of patient management. 

The eligibility for endoscopic treatment is, by far, the most important issue to be addressed. The NCCN divides the UTUC population into “favorable” or “unfavorable” according to tumor size, grade, focality, and invasiveness [11]. The EAU guidelines divide the UTUC population into “low risk” and “high risk” according to tumor focality, size, grade, variant histology, clinical stage on CT, or hydronephrosis [10]. High risk is considered an indication for RNU unless the patient has a single functioning kidney, chronic renal failure, or bilateral disease, and for whom a case-by-case strategy should be tailored. Most (92%) of our survey respondents marked low-grade pathology as an indication for endoscopic treatment, while 22.7% and 20.4% also marked high-grade pathology and cytology, respectively. Although it may be feasible to treat high-grade tumors endoscopically, it is not in line with the recommendation of either the NCCN or the EAU guidelines, or the common practice of most other urologists. Altogether, adherence to guideline tumor grade recommendations was 73.8% for endoscopic treatment. As for focality, the adherence rate to guidelines recommendations was 61.4%. Although 86.4% of survey respondents chose a single lesion as an indication for endoscopic treatment, 38.6% chose also multifocality. Although the latter is considered a high-risk criterion, some studies show that endoscopic treatment has good oncologic outcomes in multifocal disease given that it is low grade. We had earlier found that multifocality predicted time to local recurrence but not time to progression among 60 patients with low-grade UTUC and 17 patients with multifocal tumors [18]. Villa et al. [25] found that multifocality was not associated with progression among 92 patients with UTUC and 15 with a multifocal distribution. In total, the adherence rate to indications for endoscopic treatment guidelines was only 51.1% among our survey respondents.

The biopsy method is another key element in the management of UTUC since the pathological grade is at the center of decision-making for this disease while the pathological stage is more difficult to determine [26]. In total, about half of our survey respondents use forceps for performing the biopsy while the other half use baskets. The most common device was the 3 FR standard biopsy (38.6% of survey respondents). The 3 FR standard forceps require several specimens since the amount of obtainable tissue is low [27], necessitating multiple passes through the ureter/access sheath and, in our opinion, representing a clear disadvantage of this device. The flat-wire basket, which has ribbon-like strands and acts like a guillotine, was shown to be the most accurate device, with a diagnosis rate of 94% and grade determination of 93% [22], but it is used only by 13.6% of our survey respondents. Similarly, the 6 FR BIGopsy^®^ biopsy forceps (Cook Medical, Bloomington, IN, US) were shown to provide a more accurate biopsy than the 3 F standard biopsy forceps, with a diagnosis rate of 82% and 74.9%, respectively [28], but they are used by only 12.5% of our respondents. We assume that this variability is most probably related to personal preference, personal experience, and product availability. The utility of the different biopsy devices also depends upon the shape of the individual lesion. Basket devices can be used to debulk large amounts of tissue and provide an accurate diagnosis for large papillary lesions, but forceps devices may be preferable for smaller, sessile, or non-papillary lesions [28]. 

The percutaneous approach was very rarely chosen by our respondents, with 31.8% of them considering a large tumor as an indication of a percutaneous approach. This response may coincide with the use of a resectoscope by 18.2% of respondents in response to their choice of energy generator. Many reports have shown that retrograde endoscopic treatment is feasible and that it yields good oncologic outcomes even for tumors larger than 2 cm. We, too, had shown a progression-free rate of 93.2% when treating tumors that were low-grade, multifocal, and larger than 2 cm in a median follow-up of almost 2 years [13]. Scotland et al. demonstrated a 90.5% ipsilateral recurrence rate for retrograde endoscopic treatment for tumors larger than 2 cm, with an overall survival rate of 75% and a cancer-specific survival rate of 84% in a 5-year follow-up [14]. Roupret et al. considered that this approach is now rarely used as a result of the improvement in deflection mechanisms that enable retrograde treatment, as well as due to the risk of tumor seeding [29]. Still, in the rare case of difficult retrograde access, percutaneous access appears to be a viable guideline-endorsed option, and it was indeed chosen by 48.8% of our survey respondents. 

The Ho:YAG laser was the energy generator most widely used by our respondents (87.5%). This type of laser is commonly used in stone treatment as well, and therefore it is a highly available energy source, with most endourologists being very experienced with it. The Bugbee electrocautery (used by 32.9% of our respondents) is generally used when the laser fiber limits deflection and therefore access to a specific calyx [30]. This device represents a complementary tool to the laser, and it appears to be used quite commonly. The Nd:YAG laser has different physical properties than the Ho:YAG laser, penetrating much deeper into tissue and, therefore, more efficient in causing tissue necrosis, although at the price of safety and potential damage to nearby parenchyma [14,30]. It is also used only for tissue and not for stones, which can potentially limit its availability and can explain the rarity of its use (7.9%) among our respondents. The thulium laser has gained popularity in stone treatment and is effective in UTUC as well, due to its low distance of penetrance and the relatively large area of ablation [19,31]. Still, it falls behind the Ho:YAG for UTUC treatment among our respondents (22.7%). 

Jelmyto^®^ is a mitomycin-containing thermo-reversible hydrogel that is instilled retrogradely into the renal pelvis and calyces. In a recent prospective single arm clinical trial, it was proven to be an effective chemo-ablative agent for low-grade tumors smaller than 15 mm, with a 59% complete response rate [23]. Ongoing longer-term studies show that it can be used in the ablative setting, and adjuvant setting, with induction and maintenance protocols. Additionally, it can be administered retrogradely and antegradely (via a nephrostome tube) [32,33]. In our survey, 50% of respondents use Jelmyto^®^ for UTUC treatment. Contrary to the evidence that led to its FDA approval, it is being used for large tumors (10.2%), multifocal tumors (27.3%), high-grade disease (8%), any UTUC tumor (11.4%), and carcinoma in situ (6.8%). The only indication for its use that has a high level of evidence is a tumor in a complex location (18.2%). The EAU guidelines currently do not publish any recommendations regarding Jelmyto^®^. The NCCN recommendations place Jelmyto use under “endoscopic treatment”, and state that complete or near complete resection or ablation is recommended before gel administration, and that it is most suitable for a residual, low-grade, low-volume unifocal tumor in the upper urinary tract. As more experience with this treatment will be gained, more evidence of its efficacy will be published.

The NCCN and EAU guidelines do not refer in detail to disease progression after initial endoscopic management for low-risk tumors. Transformation of UTUC into a high-risk disease or a pathology with unfavorable characteristics, however, is a clear indication for radical surgery. In our survey, only 39.8% of respondents marked grade progression as an indication of referral to RNU, however, our experience as well as that of others has shown that there are cases with low-grade histology but rapid, high-volume, or frequent recurrences that some define as representing “disease progression” [13,25]. In our survey, 38.6% of respondents marked high-volume or frequent recurrences as an indication for RNU despite its not being endorsed in the NCCN and EAU guidelines.

Survey questions on patient follow-up were divided into endoscopic follow-up, cross-sectional imaging follow-up, and voiding cytology follow-up. Current guidelines do not recommend any specific protocol for endoscopic follow-up: the NCCN state that ureteroscopic follow-up or a CT urogram/magnetic resonance urogram should be performed at 3–12 months intervals after nephron-sparing surgery, while the EAU recommends that URS should be performed after 3 months for low-risk tumors. In our survey, a total of 80.7% perform a follow-up URS after 3 months, which seems like a consensus among urologists. Only 9.1% do not routinely perform follow-up URS but rather use imaging and voiding cytology tests (contrary to the NCCN recommendation but not necessarily contrary to the EAU ones). All respondents reported using cross-sectional imaging for follow-up. As for the voiding cytology test for follow-up purposes, while the NCCT recommends considering a cytology test and the EAU recommends using it only for high-risk tumors, a voiding cytology test is used by all of our survey respondents, possibly due to its noninvasive nature. 

When we looked for an association between surgeon training and experience with management strategies, we found a significant association between two areas: endoscopic follow-up regimen and fellowship (both fellowship training and fellowship type) and voiding cytology regimen with annual case volume. It is demonstrated that fellowship training was associated with a stricter voiding cytology regimen. Voiding cytology is a non-invasive inexpensive tool that may “rule-in” the presence of disease and be of certain use in the follow-up protocols. The other association that was found was the one between a stricter endoscopic follow-up regimen and an annual case volume. Since these are a result of one another, the association is somewhat obvious. 

One possible explanation for the variability in management strategies for UTUC is the rarity of the disease and the consequent lack of experience in treating it. One-half of urologic surgeons reported that the proportion of UTUC cases out of their total endoscopic cases was 0–5%. Given that almost 60% of centers in our survey have an annual volume of fewer than 300 cases, the total comes down to fewer than 15 cases per year, and about 1 case per month. Another explanation is the specific population of our respondents. The Endourologic Society was founded in the United States, and many of its members are Americans who may have limited exposure to the EAU guidelines.

Our study was the first to assess the use of endoscopic treatments among endourologists worldwide, but it is not without limitations. First, it was delivered online through the Endourological Society, which represents a specific population of urologists and not necessarily the global urologic community. Second, the survey was sent as-is, did not cover all the details of the practice patterns, and precluded the ability to expand upon or otherwise clarify responses. Third, the number of urologists that responded to the survey and the response rate were both relatively low and may limit the strength of the conclusions drawn from the provided data.

## 5. Conclusions

Our study provides insight into the practice patterns in the endoscopic treatments of UTUC. Due to the rarity of the disease, the volume of cases treated in each medical center is very low for the most part, and experience with this disease is usually limited. As expected, there is vast variability in the technical aspects of disease management. Less expected is the great variability in the practiced indications for endoscopic management and adherence to the available guidelines for managing UTUC. This demonstrates the need to further enhance the exposure to the guidelines. Future studies should explore the reasons for the limited penetration of the EAU and NCCN guidelines for UTUC management.

## Figures and Tables

**Table 1:** Upper Urinary Tract Urothelial Carcinoma Management questionnaire and responses (No multiple answers were possible unless stated otherwise).

Questions and Answers	% of Responses	Number of Responses
1. Are you fellowship trained?		
Yes	80.7	71
No	19.3	17
Total		88
2. In which specialty was the fellowship?		
Endourology	69	49
Oncology	16.9	12
Other	14.1	10
Total		71
3. Time since fellowship graduation (years)		
0–5	23.8	15
6–10	12.7	8
>10	63.5	40
Total		63
4. What is the yearly overall volume of endourology cases in your practice?		
0–100	18.2	16
100–200	29.6	26
200–300	18.2	16
300–500	17	15
>500	17	15
Total		88
5. Estimated proportion of UTUC cases out of the total endoscopic cases per year		
0–5%	56.8	50
5–10%	18.2	16
10–20%	15.9	14
20–30%	3.4	3
>30%	5.7	5
Total		88
6. Indicate your indications for UTUC endoscopic treatment (choose all that apply)		
LG UTUC	92	81
HG UTUC	22.7	20
Solitary lesion	86.4	76
Multifocal lesions	38.6	34
HG cytology	20.4	18
Total number of respondents		88
7. Proportion of percutaneous UTUC cases out of overall endoscopic UTUC cases		
0–10%	94.3	83
10–30%	5.7	5
>30%	0	0
Total		88
8. What are your indications for percutaneous UTUC treatment?		
Large tumor	31.8	28
Multifocal tumor	2.3	2
Complex approach via retrograde endoscopy	48.8	43
Personal preference	17.1	15
Total		88
9. Which device do you use to obtain a tumor biopsy?		
3 FR biopsy forceps	38.6	34
Nitinol basket	33	29
Flat wire (stainless steel) basket	13.6	12
BIGopsy^®^ (Cook Medical)	12.5	11
Piranha^®^ (Boston Scientific)	2.3	2
Total		88
10. Energy generator for UTUC treatment (multiple options may be chosen)		
Holmium (Ho:YAG) laser	87.5	77
Neodymium (Nd:YAG) laser	7.9	7
Thulium laser (Thu:YAG)	22.7	20
Diode laser		2
Electrocautery-Bugbee	32.9	29
Electrocautery—resectoscope	18.2	16
Other	20.5	18
Total number of respondents		88
11. When do you use JELMYTO^®^ adjuvant treatment? (multiple options may be chosen)		
Any UTUC tumor	11.4	10
Large tumor	10.2	9
Multifocal tumor	27.3	24
Frequent recurrences	26.1	23
Tumor in a complex location	18.2	16
HG UTUC	8	7
CIS	6.8	6
I do not use JELMYTO^®^	50	44
Total number of respondents		88
12. When do you refer UTUC patients to radical nephroureterectomy?		
Grade progression	39.8	35
Frequent recurrences	9.1	8
High-volume recurrences	29.5	26
Tumor in a complex location	5.7	5
Non-functioning kidney	15.9	14
Total		88
13. What is your endoscopy follow-up protocol for a patient who is tumor-free during the first 3 months following endoscopic treatment?		
Every 3 months for the first year and then every 6 months	52.3	46
At 3 months and then every 6 months	28.4	25
Every 6 months	7.9	7
Annually	2.3	2
Only when cytology or cross-sectional studies are suspicious of recurrence	9.1	8
Total		88
14. What is your cross-sectional imaging follow-up protocol for a patient who is tumor-free during the first 3 months following endoscopic treatment?		
Every 3 months for the first year and then every 6 months	19.3	17
At 3 months and then every 6 months	17.1	15
Every 6 months	40.9	36
Annually	22.7	20
Only when cytology is suspicious of recurrence	0	0
Total		88
15. What is your cytology follow-up protocol for a patient who is tumor-free during the first 3 months following endoscopic treatment?		
Every 3 months for the first year and then every 6 months	51.2	42
At 3 months and then every 6 months	23.2	19
Every 6 months	21.9	18
Annually	3.7	3
Total		82

**Table 2:** Association between surgeon training/experience with management patterns of UTUC.

	Fellowship Trained?	Type of Fellowship	Yearly Case Volume	Proportion of UTUC Cases
Adherence to grade guidelines	0.560	0.341	0.475	0.648
Adherence to focality guidelines	0.999	0.403	0.795	0.348
Total adherence to EAU guidelines	0.780	0.112	0.761	0.154
The proportion of percutaneous cases	0.638	0.568	0.086	0.527
Indications to percutaneous treatment	0.102	0.545	0.839	0.321
Biopsy device	0.05	0.118	0.594	0.699
Use of JELLMYTO^®^	0.991	0.931	0.399	0.671
Referral to RNU	0.435	0.371	0.079	0.270
Endoscopic follow-up	0.086	0.104	**0.001**	0.387
Cross-section imaging follow-up	0.832	0.820	0.695	0.240
Voiding cytology follow-up	**0.013**	**0.041**	0.419	0.832

**Table 3:** Association between surgeon training/experience and voiding cytology test regimen.

	Fellowship Training		Fellowship Type		
	Trained	Not Trained	Endourology	Oncology	Other
Every 3 months for the first year and then every 6 months	31 (56.4%)	11 (40.7%)	23 (51.1%)	8 (66.7%)	1 (12.5%)
At 3 months and then every 6 months	14 (25.4%)	5 (18.5%)	11 (24.5%)	3 (25%)	4 (50%)
Every 6 months	8 (14.6%)	10 (37.1%)	9 (20%)	1 (8.3%)	3 (37.5%)
Annually	2 (3.6%)	1 (3.7%)	2 (4.4%)	0	0
	55	27	45	12	8

**Table 4:** Association between annual case volume and endoscopic follow-up regimen.

	0–100	100–200	200–300	300–500	>500
Every 3 months for the first year and then every 6 months	3 (18.8%)	13 (50%)	10 (62.5%)	10 (66.7%)	10 (66.7%)
At 3 months and then every 6 months	6 (37.5%)	8 (30.8%)	1 (6.2%)	5 (33.3%)	5 (33.3%)
Every 6 months	4 (25%)	0	3 (18.8%)	0	0
Annually	1 (6.2%)	1 (3.8%)	0	0	0
Only when voiding cytology is suspicious for recurrence	2 (12.5%)	4 (15.4%)	2 (12.5%)	0	0
	16	26	16	15	15

## Data Availability

Data is contained within the article or Appendix A.

## Appendix A. Upper Urinary Tract Urothelial Carcinoma Management Questionnaire

1. Are you fellowship trained?
  - Yes
  - No

2. In which specialty was the fellowship?
  - Endourology
  - Oncology
  - Other

3. When did you graduate from your fellowship?
4. What is the yearly overall volume of endourology cases in your practice?
  - 0–100
  - 100–200
  - 200–300
  - 300–500
  - 500<

5. Estimated proportion of UTUC cases out of the total endoscopic cases per year:
  - 0–5%
  - 5–10%
  - 10–20%
  - 20–30%
  - 30%<

6. Mark your indications for UTUC endoscopic treatment (choose all that apply):
  - LG UTUC
  - HG UTUC
  - Solitary lesion
  - Multifocal lesions
  - HG cytology

7. What is the proportion of percutaneous UTUC cases out of all endoscopic UTUC cases?
  - 0–10%
  - 10–30%
  - 30%<

8. What are your indications for percutaneous UTUC treatment?
  - Large tumor
  - Multifocal tumor
  - Complex approach via retrograde endoscopy
  - Personal preference

9. Which device do you use to obtain a tumor biopsy?
  - 3 FR biopsy forceps
  - Nitinol basket
  - Flat wire basket
  - BIGopsy^®^
  - Piranha^®^

10. Which energy generator do you use for UTUC treatment? (multiple options may be chosen):
  - Holmium laser
  - Neodymium laser
  - Thulium laser
  - Electrocautery—Bugbee
  - Electrocautery—resectoscope

11. When will you use JELMYTO^®^ adjuvant treatment? (multiple options may be chosen):
  - Any UTUC tumor
  - Large tumor
  - Multifocal tumor
  - Frequent recurrences
  - Tumor in a complex location
  - HG UTUC
  - CIS
  - I will not use JELMYTO^®^

12. When do you refer UTUC patients to radical nephroureterectomy?
  - Grade progression
  - Frequent recurrences
  - High volume recurrences
  - Tumor in a complex location
  - Non-functioning kidney

13. What is your endoscopy follow-up protocol for a patient who is tumor-free during the first 3 months following endoscopic treatment?
  - Every 3 months for the first year and then every 6 months
  - At 3 months and then every 6 months
  - Every 6 months
  - Annually
  - Only when cytology is suspicious for recurrence

14. What is your cross-sectional imaging follow-up protocol for a patient who is tumor-free during the first 3 months following endoscopic treatment?
  - Every 3 months for the first year and then every 6 months
  - At 3 months and then every 6 months
  - Every 6 months
  - Annually
  - Only when cytology is suspicious for recurrence

15. What is your cytology follow-up protocol for a patient who is tumor-free during the first 3 months following endoscopic treatment?
  - Every 3 months for the first year, and then every 6 months
  - At 3 months, and then every 6 months
  - Every 6 months
  - Annually
